# Single-trial lie detection using a combined fNIRS-polygraph system

**DOI:** 10.3389/fpsyg.2015.00709

**Published:** 2015-06-02

**Authors:** M. Raheel Bhutta, Melissa J. Hong, Yun-Hee Kim, Keum-Shik Hong

**Affiliations:** ^1^Department of Cogno-Mechatronics Engineering, Pusan National UniversityBusan, South Korea; ^2^FIRST 5 Santa Clara CountySan Jose, CA, USA; ^3^Department of Physical and Rehabilitation Medicine, Center for Prevention and Rehabilitation, Heart Vascular and Stroke Institute, Samsung Medical Center, Sungkyunkwan University School of Medicine, Samsung Advanced Institute of Health Sciences & Technology, Sungkyunkwan UniversitySeoul, South Korea; ^4^School of Mechanical Engineering, Pusan National UniversityBusan, South Korea

**Keywords:** deception, single-trial lie detection, combined fNIRS-polygraph system, hemoglobin change, physiological responses, linear discriminant analysis

## Abstract

Deception is a human behavior that many people experience in daily life. It involves complex neuronal activities in addition to several physiological changes in the body. A polygraph, which can measure some of the physiological responses from the body, has been widely employed in lie-detection. Many researchers, however, believe that lie detection can become more precise if the neuronal changes that occur in the process of deception can be isolated and measured. In this study, we combine both measures (i.e., physiological and neuronal changes) for enhanced lie-detection. Specifically, to investigate the deception-related hemodynamic response, functional near-infrared spectroscopy (fNIRS) is applied at the prefrontal cortex besides a commercially available polygraph system. A mock crime scenario with a single-trial stimulus is set up as a deception protocol. The acquired data are classified into “true” and “lie” classes based on the fNIRS-based hemoglobin-concentration changes and polygraph-based physiological signal changes. Linear discriminant analysis is utilized as a classifier. The results indicate that the combined fNIRS-polygraph system delivers much higher classification accuracy than that of a singular system. This study demonstrates a plausible solution toward single-trial lie-detection by combining fNIRS and the polygraph.

## Introduction

Accurate and reliable lie detection represents an intriguing challenge for experts in various scientific fields. The objective of this paper is to enhance the classification accuracy in single trial lie detection paradigm by combining a functional near-infrared spectroscopy (fNIRS) and autonomic data (i.e., electrodermal and respiratory activities). A polygraph, which measures several physiological parameters in the body including respiration, skin conductance, blood pressure, and pulse rate, is widely employed in lie-detection settings (Jung, 1919; Mohamed et al., 2006; Matsuda et al., 2013; Walczyk et al., 2013).

Many researchers support the hypothesis that direct measurement of brain functions might enable more complete understanding of deception and, therefrom, more accurate and consistent detection of lies. Based on this hypothesis, many researchers have investigated various neurophysiological signals for possible use in lie-detection applications. Among these signals are event-related potentials (ERPs), which are acquired by electroencephalography (EEG) from the scalp (Duncan et al., 2009; Rosenfeld et al., 2012). ERPs are used mostly to test for knowledge about the crime details known only to the criminal involved (Farwell and Donchin, 1991). This type of test is commonly known as a guilty knowledge test (GKT) or concealed information test (CIT) (Zhao et al., 2012; Farahani and Moradi, 2013; Rosenfeld et al., 2013). EEG, with its high temporal resolution, detects brain signals very quickly (Turnip et al., 2011; Cantilena et al., 2012; Soekadar et al., 2014), but the main disadvantage is its poor spatial resolution. The other technique widely used to detect brain areas that are activated in the course of deception is functional magnetic resonance imaging (fMRI). fMRI, with its high spatial resolution relative to that of EEG (Spence et al., 2004; Yuan and Ye, 2013; Liang et al., 2014), can easily localize changes in the regional cerebral blood flow (rCBF). A comprehensive review of fMRI-based deception decoding has found the current fMRI technique to be limited due to high cost of its scanners, bulky size, and its high sensitivity to motion artifacts (Farah et al., 2014).

Researchers therefore have begun to explore another brain-imaging technique, fNIRS, which measures brain activity through hemodynamic responses associated with neuron behavior (Kochel et al., 2011; Kamran and Hong, 2013; Santosa et al., 2013; Hong and Nguyen, 2014; Tempest et al., 2014; Naseer and Hong, 2015b), in doing so it provides both topographic (Wolf et al., 2007) and tomographic brain images (Barbour et al., 2001): A topographic image is to depict the brain activities on the brain surface, whereas a tomographic image is to characterize the internal difference in the brain. In the case of fNIRS, the hemodynamic responses in association with the brain activities can be topographically mapped on the brain surface (i.e., intensity map), whereas the dynamics of the vascular responses during the neural activation can be tomographically reconstructed in the 3D space (Barbour et al., 2001).

Near-infrared (NIR)-spectrum light takes advantage of a 650 ~ 1000 nm optical window in which skin, tissue, and bone are mostly transparent to NIR light, but wherein oxy-hemoglobin (HbO) and deoxy-hemoglobin (HbR) are strong light absorbers. fNIRS provides a better temporal/spatial resolution trade-off than does EEG or fMRI. A comprehensive comparative evaluation of the respective features of fNIRS and fMRI indicated that fNIRS has a much greater potential for psychiatric and neurological applications, owing specifically to its better portability, simplicity, and insensitivity to motion artifacts (Irani et al., 2007). fNIRS therefore has been used in a great variety of fields, including neuroscience (Hu et al., 2011), sports medicine (Quaresima et al., 2003), behavioral studies (Roos et al., 2011; Santosa et al., 2014), clinical medicine (Taillefer and Denault, 2005), pediatrics (Lanfranconi et al., 2014), and brain-computer interface (BCI) (Fazli et al., 2012; Naseer and Hong, 2013, 2015a; Naseer et al., 2014). Recently, small, portable and cost-effective fNIRS systems have extended the application of this modality to outdoor activities such as exercise or those conducted for rehabilitation purposes (Muehlemann et al., 2008; Kim et al., 2011; Bhutta et al., 2014; Khan et al., 2014; Piper et al., 2014).

To date, only very limited research work has been undertaken in the field of fNIRS-based deception decoding (Tian et al., 2009; Hu et al., 2012; Ding et al., 2013, 2014; Sai et al., 2014). In fact, no study has yet utilized or compared the fNIRS signals with physiological changes of a human body in a deception-decoding-experimental setting. There are only a few studies that have used physiological signals in parallel with fNIRS measurement (Falk et al., 2011; Zimmermann et al., 2013), and most of those were conducted only for BCI purposes.

In the present study, we performed offline single-trial classification of the truthful and deceptive responses of healthy male subjects. More specifically, we addressed the issues of (i) how truthful and deceptive responses are classified based on the fNIRS data collected from the prefrontal cortex (PFC), and (ii) how the inclusion of body physiological parameters including respiration, electrodermal activity (EDA) and body movement can affect classification accuracy. Linear discriminant analysis (LDA) was used to distinguish truthful from deceptive responses based on the fNIRS-determined mean and slope values of HbO combined with polygraph-based respiration and EDA results. The results obtained in this study demonstrate that the classification accuracy attained by the combined fNIRS-polygraph system was superior to that of either single system. To the best of our knowledge, this is the first time that polygraph has been combined with the fNIRS system in the lie-detection context.

## Materials and methods

### Subjects

The experimentation was performed with 16 healthy male subjects (mean age: 31.2 ± 3.2). All had normal or corrected-to-normal vision. Among these 16 subjects, 13 were right handed and 3 were left handed. None of the subjects had a history of any neurological or psychiatric disorder. The Institutional Review Board of Pusan National University had approved this work. The subjects' written consent was obtained before the experiment, and the ethical standards of the latest Declaration of Helsinki were observed during the experiment.

### Experimental procedure

**Figure 1:**
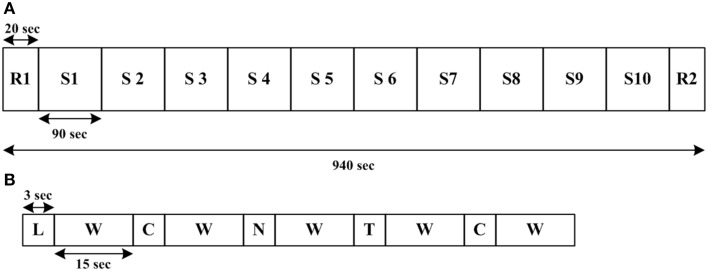
**Experimental procedure. (A)** Total 10 sessions with two rest sessions: R for rest and S for session, **(B)** a sample session: L for lie, W for wait, C for control, N for neutral, and T for true.

The experimental paradigm in this study is a modified version of the mock theft scenario presented by Tian et al. (2009), according to which the subject, left alone in a room, was instructed to steal either a 5000 KRW note or a 10,000 KRW note from the drawer of a table and keep it in his pocket. The subject was then brought out of the room and taken to another room for questioning. The interrogator was totally unaware of the stolen note. Prior to the start of the formal deception experiment, each subject was given a brief description of the experiment, and a practice session was performed to confirm his complete understanding of the experimental procedure. The deception experiment required the subject to answer three types of questions (see Appendix in Supplementary Materials): “True/Lie” questions related to the stolen note and the note left in the drawer, neutral questions were general questions, answers to which were objectively “yes” or “no,” and control questions relevant to the subject's personal life and some minor transgressive acts. The subject was instructed clearly that he has to deny the possession of the stolen note and had to answer a lie for only the questions related to the stolen note. In this study we only discriminate between the lie and the truth response of the subjects. The neutral and control questions were asked just to keep the subject active. A total of 10 question sessions, each session comprising five random questions, were presented on a computer screen. In each session, one true, one lie, one neutral, and two control questions were posed. Each question was displayed for about 3 s, and the subject had to answer mentally as soon as he finished the reading. After each answer, a 15 s wait period was given for the settling of hemodynamic signals. There were two rest periods, each of 20 s, given to the subject: one in the start of the experiment and the other one at the end of the experiment. In this study, six of the subjects took the 5000 KRW note, and the other 10 took the 10,000 KRW note. Figure 1 illustrates the experimental procedure and a sample experimental trial.

### Data acquisition

**Figure 2:**
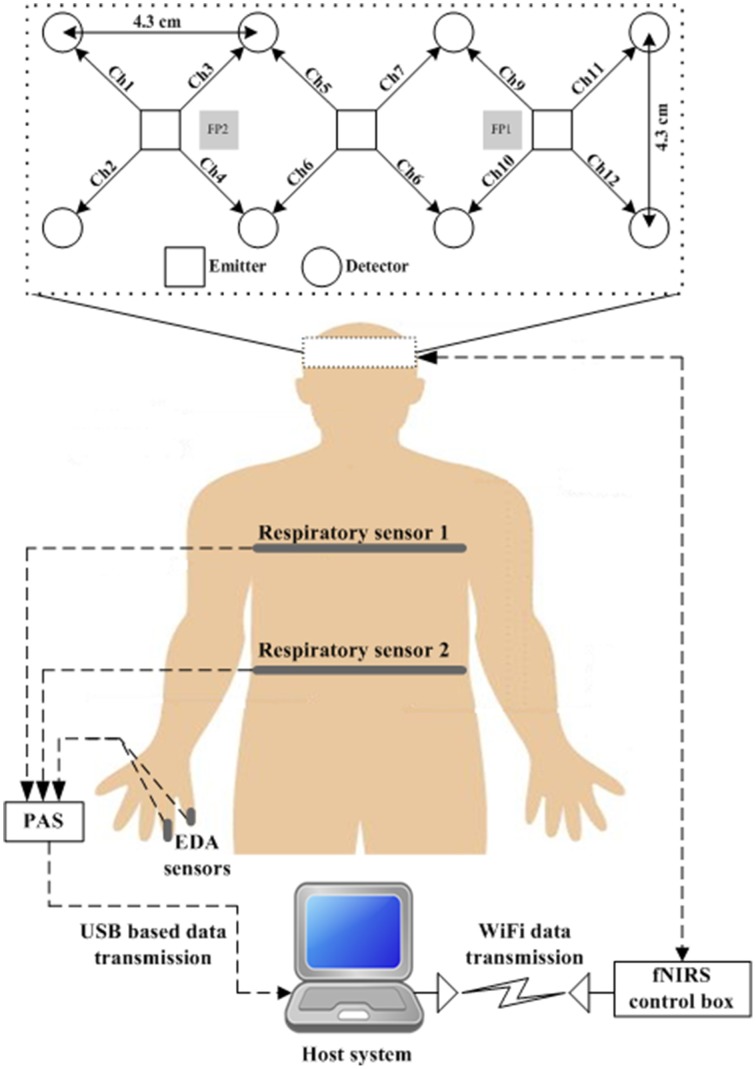
**Block diagram of measurement setup**. The physiological signals (respiration and EDA) are transmitted to the Paragon Acquisition System (PAS), in which they are converted to digital data and then sent to the host system through a USB cable. The fNIRS probe is connected to the control box, which converts the analog light-intensity signals to digital signals and then transmits them to the host system by WiFi wireless transmission.

A multi-channel continuous-wave imaging system developed by the authors (Bhutta et al., 2014) was used to acquire the brain signals. The optical probe of the fNIRS system was positioned on the forehead of the subject such that the emitters of the probe were in parallel to the FP1 and FP2 locations of the international 10–20 system. The hair was brushed backward to clear the forehead for attaching the flexible probe. The emitters and detectors were arranged to have good contact with the scalp and were then fixed to the subject's head with self-adhesive bandages. The probe configuration includes three near-infrared light-emitting diodes (LEDs) as emitters (each of which can emit three wavelengths: 640, 700, and 910 nm) and eight Si-photodiodes as detectors, see Figure 2. The emitters and detectors were systematically positioned within a 5 × 14 cm^2^ area according to the source-to-detector distance of 3 cm. Each LED was turned on and off sequentially, and the light diffused through the cortical region was detected at the nearest detectors. A total of 36 light-intensity signals (3 LEDs × 3 wavelengths × 4 neighboring detectors) were acquired at a sampling rate of 3.8 Hz. A Velcro band kept the probe in the required position during the experiment.

To measure the physiological conditions of a human body (i.e., respiration, body movement, and skin conductance), we used the Paragon Acquisition System (PAS) developed by Limestone Technologies (Canada). The transducers were positioned as per the guidelines posted at the Limestone Technology website^1^. A Piezo Electronic StingRay_SE CM seat sensor, positioned on the chair where the subject would naturally rest most of his weight, recorded the subject's body movement. For two pneumatic respiration transducers provided in the PAS, one was affixed beneath the last rib and another one near the top rib of the subject. Two EDA 24 K gold plated metal electrodes were used for maximal sensitivity to monitor the skin conductance or EDA. These electrodes were attached with a Velcro ring to the last two fingers of the subject's right hand. Micro-volt voltage was continuously applied between the electrodes, and the inter-electrode current was measured to acquire the EDA signal. All of these transducers were connected to the PAS via metal connectors, and the PAS was connected to the host PC through a USB cable. Figure 2 provides a complete block diagram of the system indicating not only the sensor positions but also the connections between the sensors and the host system.

### Data processing

**Figure 3:**
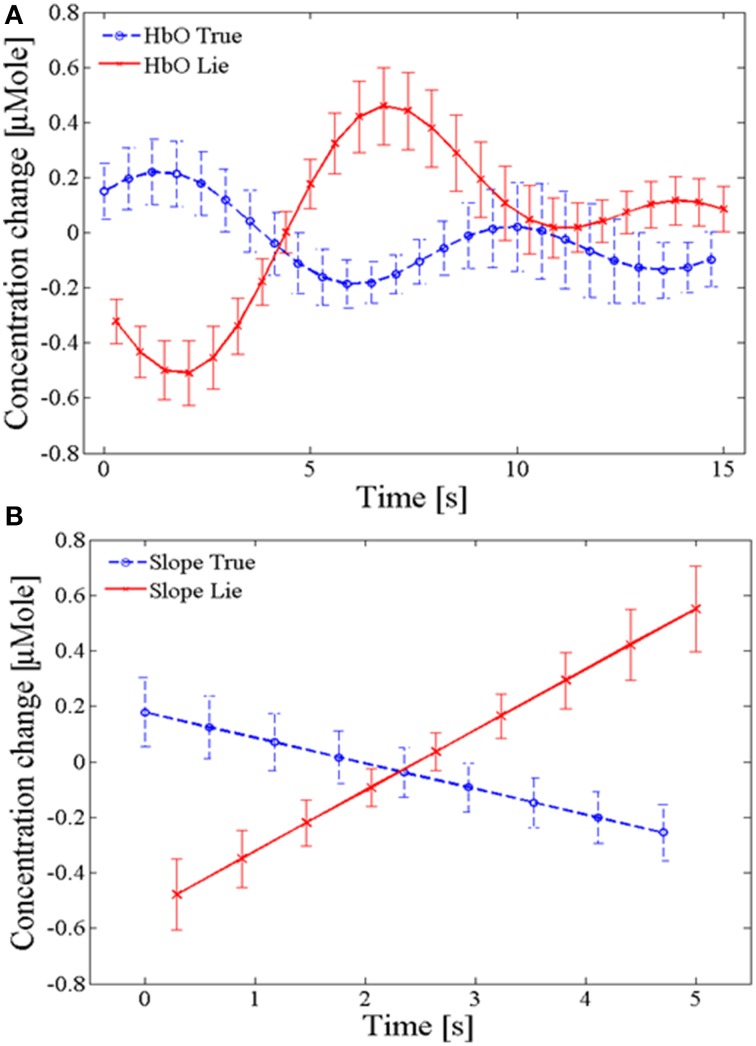
**Averaged oxy-hemoglobin responses. (A)** Comparison of HbO means for true and lie responses during the 15 s wait period in Figure 1B, **(B)** comparison of HbO slopes during the early 2~7 s interval out of the 15 s wait period. Data were averaged over all 10 trials and 16 subjects (i.e., total 160 responses for each lie and true).

The signals from both measuring systems (fNIRS, polygraph) were imported and further analyzed offline using MATLAB 7.9.0 (MathWorks, USA). The data were stored in a host-computer text file in the form of digitized raw intensity values from the fNIRS system. From these raw intensity values, the changes in optical density, Δ*OD*, could be calculated at each discrete time *k* as

(1) $$\Delta OD(k;\lambda )=\log_{10}\frac{I_{0}(\lambda )}{I(k;\lambda )}=ld(\lambda )\Delta \mu_{a}(k;\lambda )$$

where *I* is the intensity of the detected light, *I*_0_ is the intensity of the incident light, *d* is the differential pathlength factor (DPF), *l* is the distance between the emitter and the detector, and Δμ_*a*_ is the absorption change of the tissue. The changes of oxy-hemoglobin (Δ*c_HbO_*) and deoxy-hemoglobin (Δ*c_HbR_*) were measured using the modified Beer-Lambert law (Kocsis et al., 2006) as

(2) $$\begin{array}{l} \left[\begin{array}{c} \Delta c_{\text{HbO}}(k)\\ \Delta c_{\text{HbR}}(k) \end{array}\right]={\left[\begin{array}{cc} ld^{\lambda_{1}}\alpha_{\text{HbO}}^{\lambda_{1}} & ld^{\lambda_{1}}\alpha_{\text{HbR}}^{\lambda_{1}}\\ ld^{\lambda_{2}}\alpha_{\text{HbO}}^{\lambda_{2}} & ld^{\lambda_{2}}\alpha_{\text{HbR}}^{\lambda_{2}} \end{array}\right]}^{-1}\\ \;\times \left[\begin{array}{c} \Delta OD_{1}(k,\lambda )\\ \Delta OD_{2}(k,\lambda ) \end{array}\right], \end{array}$$

with λ_1_ = 640 nm, λ_2_ = 910 nm, *d*^λ 1^ = 6.63, and *d*^λ 2^ = 2.765 (Scholkmann and Wolf, 2013), according to the values for the wavelength-dependent absorption coefficients α_HbO_, α_HbR_ taken from the website of the University College London (Department of Medical Physics and Bioengineering, the title of the website: Specific Extinction Spectra of Tissue Chromophores)^2^. fNIRS, in detecting the hemodynamic response, picks up the physiological noises of respiration, pulse and low-frequency Mayer waves. To remove such noises, a second-order low-pass filter with a cutoff frequency of 0.15 Hz was used. Figure 3A shows the plots of averaged HbO signals for “true” and “lie” responses with its standard deviation (SD) and Figure 3B shows the averaged slope of the HbO signal for 2 ~ 7 s window out of the 15 s wait period with its SD. The data were averaged over all 10 trials and 16 subjects.

**Figure 4:**
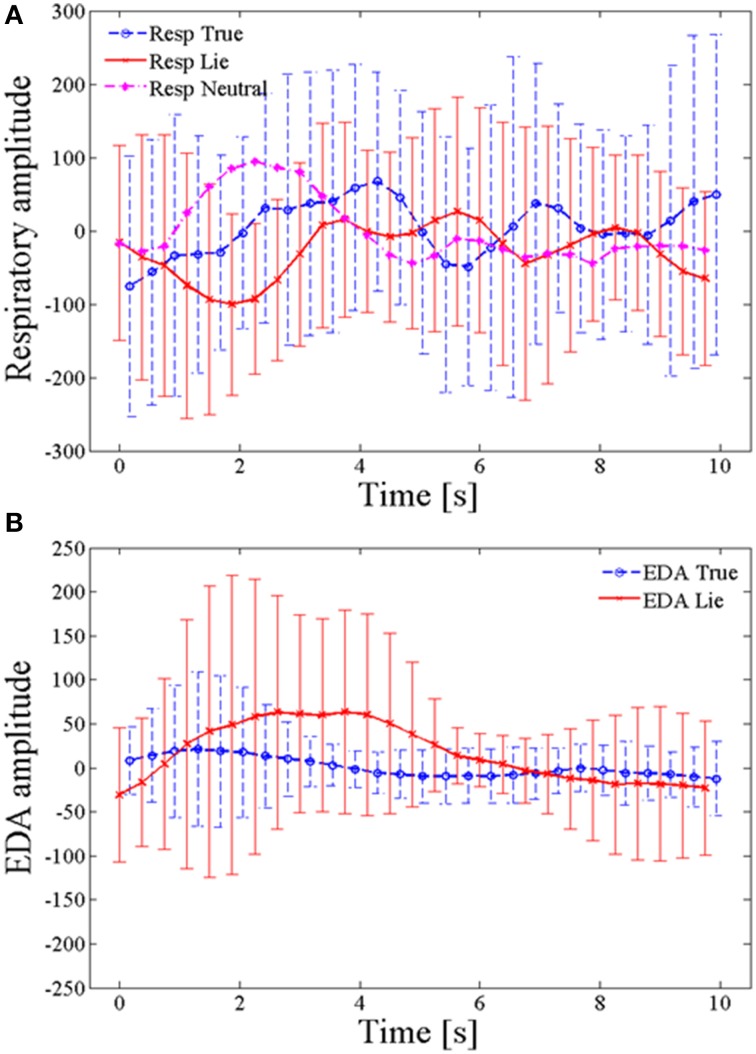
**Averaged polygraph-based physiological responses. (A)** Averaged respiratory responses for lie, true, and neutral questions, **(B)** averaged EDA responses for lie and true. Data were averaged over all 10 trials and 16 subjects.

The PAS data were stored in a text file in the form of digitized values from the corresponding transducers. For simplicity and uniformity, the saved data were then normalized to a baseline for all of the transducers. Figure 4 depicts the averaged data for “true” and “lie” physiological responses with their SD, (Figure 4A: respiration amplitude, Figure 4B: EDA amplitude), recorded by the polygraph. The data were averaged over all 10 trials and 16 subjects.

### Classification

In this study, LDA was used to distinguish “lie” from “true” responses because of its simplicity and execution speed. LDA is a linear classifier that discriminates between the different classes of data based on the hyper-planes (Lotte et al., 2007). The separating hyper-plane is designed in such a way that it should minimize the interclass variance and maximize the distance between the class means.

Classification was performed on the data segments acquired during the wait period after each true and lie response. For classification purpose we used 20 points (10 true and 10 lie) for each subject, as shown in Figure 1, there are 10 session of our experiment and each session has at least one true and one lie question in it. The classification accuracies were calculated using 10 runs of a ten-fold cross-validation, that is, one truth/lie trial was selected for the testing purpose whereas the classifier was trained on the remaining nine trials. This process was repeated for all the trials. The classification accuracies were determined with the error averaged over all training/testing combinations.

In this study, only HbO signals were considered for classification purposes of fNIRS data. Signal mean (SM) and signal slope (SS) of HbO are used as classification features. A grand average of HbO was taken over all 12 channels. The SS value for each wait period was determined by fitting a line to all the data points during the 2 ~ 7 s window of the wait period, using the linear regression. Hong et al. (2015) has shown that SM and SS give the best result when a window of 2 ~ 7 s after the stimulus is used for getting SM and SS. The SM value for each wait period was determined by averaging the data points in the respective time windows.

In this study, respiration and EDA signals were used for classification of polygraph data. The motion sensor values were excluded from the classification process owing to the lack of significant difference between the recorded “true” and “lie” response readings. For the polygraph data, only the first 7 s of each wait period after the true and lie questions were analyzed. As two respiratory sensors were used, the average value over both sensors was taken. The mean values of respiration and EDA signals were used as classifier features for the polygraph data. The mean values were calculated by averaging the data points during the 7 s time window.

After calculating the classification results for individual modalities (fNIRS, polygraph), a combined classification accuracy was also calculated by using LDA as meta-classifier. Then, the features used in individual calculations were combined to get the combined classification accuracy.

## Results

**Figure 5:**
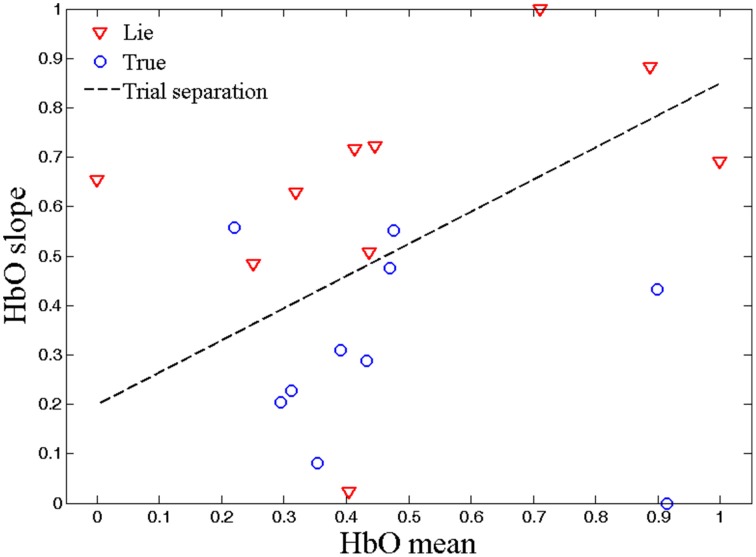
**fNIRS data classification**. NIRS data classification for lie and true responses using HbO mean and HbO slope (for 2–7 s interval, Subject 15).

**Figure 6:**
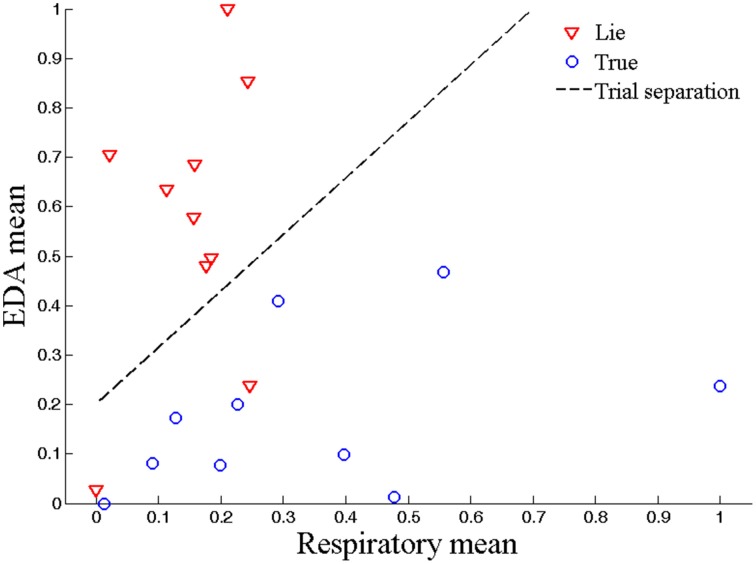
**Polygraph data classification**. Polygraph data classification for lie and true responses using respiration mean and EDA mean (for 1–7 s interval, Subject 15).

Figure 5 shows the two classes (true and lie) obtained after classification of the HbO mean and HbO slope data for the true and lie data of Subject 15. Figure 6 shows the same two classes after classification of the same subject's respiration and EDA data values. It can be seen from Figures 5, 6 that the feature values were scaled between 0 and 1 using the equation below.

(3) $$s^{\prime }=\frac{s-\min(s)}{\max(s)-\min(s)}$$

where *s* denotes the original data for each feature, *s*' denotes the scaled value of the respective feature, min(*s*) and max(*s*) denote the minimum and maximum values in the respective feature set.

**Figure 7:**
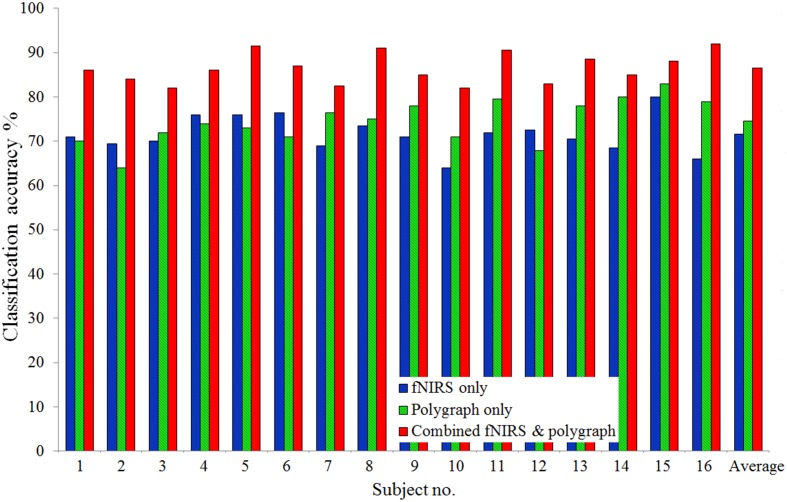
**Comparison**. Comparison of classification accuracies of individual modalities and the combined system.

To compare the results obtained by the combined system and individual systems, the true and lie responses were classified once again using four features (HbO mean, HbO slope, respiration, and EDA mean values): Figure 7 compares the classification accuracies of individual systems and the combined system. As averaged over all 16 subjects, the classification accuracies obtained from individual fNIRS and polygraph modalities were 71.6 and 74.5%, respectively, whereas with the combined fNIRS-polygraph system, a very considerable improvement, to 86.5%, was achieved. The classification accuracies obtained by the combined fNIRS-polygraph system were compared with those obtained by the individual systems using the non-parametric Mann–Whitney *U*-test. The results for the combined system vs. fNIRS alone were *U*_(16)_ = 256 and *p* < 0.0001, whereas those for the combined system vs. the polygraph alone were *U*_(16)_ = 252.5 and *p* = 0.0001.

## Discussion

The polygraph is widely used in interrogations (i.e., criminal investigations) and office-interview settings to detect lies (Ginton, 2013). The major physiological responses measured are respiration, heart rate, skin conductance, body movement, and blood pressure. In this study, a commercially available polygraph machine, the Paragon Acquisition System (PAS), was used to detect the physiological signals. Respiration, electrodermal activity, and body movement were measured from the respective measuring points shown in Figure 2. The body movement data were excluded from the analysis, owing to the lack of difference in detected data. The reason for this might have been the fact that for the fNIRS measurements, the subjects were asked to remain still and not move their body, as this can generate noise in fNIRS data. The measured signals from the respiration and EDA transducers were fed to the PSA, which converted them to digital and then sent them to the host system using a wired connection.

Figure 4 shows a clear and significant difference between the respiration and EDA signals corresponding to true and lie responses. It can be seen from the plot that in the first few seconds of the wait period the respiration amplitude for lie is relatively lower than the amplitude during the truth and the EDA signals goes high during the first few seconds of the lie condition. These findings are in accordance with the previous literature (Matsuda et al., 2011). These are only some of the physiological measures for lie detection; nevertheless, scientists believe that provided the brain signals activated during the deception process can be clearly observed, understanding the deception phenomenon and detecting them are easily done. In the present study, for direct detection of brain signals, the fNIRS system was used to acquire hemodynamic signals from the brain's PFC. There are multiple areas in the brain that are involved in lying but the previous studies have already established that the PFC is activated when an individual practices deception (Christ et al., 2009; Hu et al., 2012; Ding et al., 2014; Farah et al., 2014; Guhn et al., 2014; Sai et al., 2014). Figure 3 shows the clear and significant difference between the HbO signals corresponding to lie and true responses. It serves to demonstrate that true and lie responses can be separated from each other based on the respective relevant hemodynamic responses from the PFC.

The classification was performed using LDA to distinguish HbO signals (as fNIRS data features) along with respiration and EDA signals (as polygraph data features). Figures 5, 6 show the two different classifications (true and lie) obtained after classification of the fNIRS data and polygraph data, respectively, for the same subject (Subject 15). The separability of the hemodynamic and physiological responses arising during the lie and true responses is clear.

This is the first study in which fNIRS and polygraph are combined to detect deception. As noted above, fNIRS detects the brain activity occurring during the deception process, while polygraph detects the physiological response from the body. It was assumed that by combining the two methodologies, system performance would be improved and, in fact, the data collected during this study proved the correctness of the assumption. Figure 7 compares the classification accuracies of the single measuring systems and that of the combined fNIRS-polygraph system. The averaged classification accuracies of individual fNIRS and polygraph were 71.6 and 74.5%, respectively; but that of the combined fNIRS-polygraph system, remarkably, was 86.5%. It was observed that, in some trials, fNIRS was unable to detect the deception but PAS was able to detect it and vice versa. These results prove emphatically that the combined system is more efficient in discriminating between true and lie responses.

As polygraph is widely used in criminal investigations and court settings, the observed 74.5% classification accuracy of polygraph seems rather weak. The reason for this low accuracy can be because, the used questions from Tian et al. (2009) in this paper were not so challenging. Probably, in the criminal investigations, the questions are well arranged in relation to the crime details involved. Recently, Meijer et al. (2014) published a paper, in which the authors have conducted a meta-analysis for the verification of different deception decoding paradigms using four physiological measures: skin conductance, respiratory response, heart rate, and P300 component of EEG. They have conducted their analysis over more than hundred published studies from 1970 ~ 2013. The average correct detection rate for guilty and innocent subjects using skin conductance and respiratory response ranges from 80 to 85% for all studies. The 74.5% accuracy found in our study is comparable to the average obtained by the above mentioned study. An important point should also be noted that, the results of this study cannot be directly compared with the results of previous studies because the classification accuracies reported in this study refer to differentiation between responses within individuals, whereas most previous studies deal with differentiating between deceptive and truthful individuals or groups.

All of the data analysis was performed offline, due to the limitation of the PAS, which provides data only after completion of an experiment. This limitation will be overcome by using a system that can provide physiological measurements in real time. Also, the present experiments were performed only with male subjects aged 27–34, though differences in the hemodynamic responses of females and older individuals relative to those of young males has already been reported (Anokhin et al., 1996; May et al., 2014; Zhou et al., 2014). One additional factor that should be noted is that all of our subjects were university graduate students with no criminal background. Considering that lie-detection systems are used mostly in investigations of criminals, the great majority of whom are not well educated and, often, inured to the criminal-investigative process, further research will have to be done to test the performance of our proposed combined fNIRS-polygraph system for real criminal-investigative contexts.

## Conclusions

In this study, a combined fNIRS-polygraph system for single-trial lie detection was compared with each fNIRS and polygraph system. The fNIRS system decodes deception based on the hemodynamic changes of oxy-hemoglobin measured at the prefrontal cortex, and the polygraph operates on the basis of physiological responses from the body such as respiration and electrodermal activities. Both modalities were time-synchronized, and the data were classified first separately for each system and then combined using the linear discriminant analysis as a classifier. The classification accuracy achieved by the combined system was much higher than those achieved by the single systems. These results indicate that the combined fNIRS-polygraph system offers a great potential for application to real-life lie-detection contexts.

## Author contributions

MB performed the experiment and carried out the data processing, MH and YK reviewed the manuscript and suggested a number of critical methodologies to improve the manuscript, and KH supervised the research and corrected the entire manuscript. All of the authors read and approved the final manuscript.

### Conflict of interest statement

The authors declare that the research was conducted in the absence of any commercial or financial relationships that could be construed as a potential conflict of interest.

## Footnotes

^1^Limestone technologies website. http://www.limestonetech.com/index.cfm/home/

^2^UCL Department of Medical Physics and Bioengineering, “Specific extinction spectra of tissue chromophores.” http://www.medphys.ucl.ac.uk/research/borl/research/NIR_topics/spectra/spectra.htm/

## Abbreviations

BCI: Brain-computer interface
DPF: Differential pathlength factor
EDA: Electrodermal activity
EEG: Electroencephalography
ERP: Event-related potentials
fMRI: Functional magnetic resonance imaging
fNIRS: Functional near-infrared spectroscopy
GKT: Guilty knowledge test
HbO: Oxy-hemoglobin
HbR: Deoxy-hemoglobin
LDA: Linear discriminant analysis
LED: Light-emitting diode
NIR: Near-infrared
PAS: Paragon Acquisition System
PFC: Prefrontal cortex
rCBF: Regional cerebral blood flow
SM: Signal mean
SS: Signal slope.
