# Supplementary material for: Single-trial lie detection using a combined fNIRS-polygraph system
Source: Front Psychol. 2015 Jun 2;6:709. doi: 10.3389/fpsyg.2015.00709 (PMC4451253; doi:10.3389/fpsyg.2015.00709)
Supplement: Supplementary file 1 [file DataSheet1.DOCX]

**Appendix: Questions asked during the experimental procedure**

**“5,000 KRW note” questions**

1. Did you steal the 5,000 KRW note from the drawer?
2. Is the stolen 5,000 KRW note in your pocket?
3. Was the 5,000 KRW note stolen?
4. Do you have the stolen 5,000 KRW note?
5. Did you leave the 5,000 KRW note in the drawer?
6. Did you take someone else’s 5,000 KRW note from the drawer?
7. Do you know who took the 5,000 KRW note from the drawer?
8. Is the 5,000 KRW note still in the drawer?
9. Did you place the stolen 5,000 KRW note in your pocket?
10. Are you hiding the stolen 5,000 KRW note?

**“10,000 KRW note” questions**

1. Did you hide the 10,000 KRW note?
2. Did you put the stolen 10,000 KRW note in your pocket?
3. Do you know who took the 10,000 KRW note?
4. Was the 10,000 KRW note moved from the drawer?
5. Are you hiding the stolen 10,000 KRW note?
6. Is the stolen 10,000 KRW note in in your possession?
7. Did you leave the 10,000 KRW note in the drawer?
8. Did you take a 10,000 KRW note that is not yours?
9. Did you not steal the 10,000 KRW note from the drawer?
10. Is the stolen 10,000 KRW note still in your pocket?

**Control questions**

1. Right now are you living in Busan?
2. Are you sitting down?
3. Are you sitting on a chair?
4. Do you like noodles?
5. Have you ever committed a crime?
6. Do you like to swim?
7. Do you obey every traffic law?
8. Are you under the age of 50?
9. Right now are you in Korea?
10. Have you ever forged a signature?
11. Do you belong to Korea?
12. Have you ever been to the USA?
13. Can you speak English?
14. Do you like movies?
15. Do you have a dog?
16. Have you ever been arrested?
17. Are you a citizen of Korea?
18. Have you ever cheated on a test?
19. Are you awake?
20. Have you ever faked an illness?

**Neutral questions.**

1. Is this the month of April?
2. Is it Thursday today?
3. Is it 2014?
4. Is Busan a part of South Korea?
5. Is it the spring season?
6. Is it daytime?
7. Is it nighttime?
8. Are lights ON in the room?
9. Is it raining outside?
10. Is it sunny today?
